# Les professionnels de santé et la COVID-19 au Maroc: accident de travail ou maladie professionnelle?

**DOI:** 10.11604/pamj.2021.39.283.30471

**Published:** 2021-08-31

**Authors:** Meryem Bouchalta, Ahmed Belhousse, Ghizlane Mouttarazouk

**Affiliations:** 1 Unité de Médecine Légale, Centre Hospitalier Universitaire Ibn-Sina, Rue Mfadel Cherkaoui, Souissi, Rabat, Maroc,; 2 Service de Médecine Légale, Centre Hospitalier Universitaire Ibn-Sina Ibn-Rochd, Quartier des Hôpitaux, Casablanca, Maroc

**Keywords:** COVID-19, santé au travail, droit, maladie professionnelle, accident de travail, Maroc, COVID-19, occupational health, law, occupational disease, work-related accident, Morocco

## Abstract

L´abnégation et le sens de l´engagement au devoir sont des principes éthiques fondamentaux de la profession médicale. Depuis le début de la crise sanitaire, le personnel soignant fut le premier rempart contre la propagation du coronavirus, par conséquent, la catégorie professionnelle la plus exposée aux risques de contamination. A cet égard, dans un communiqué datant du 23 mars 2020, l´Organisation mondiale de la santé a recommandé la prise en charge -au titre de législation professionnelle- de la maladie provoquée par la COVID-19 pour les salariés du secteur médical, mais également ceux de tous les secteurs exposés aux risques de contamination. Au Maroc, le ministère de la Santé a publié le 6 avril, sur son site officiel, un communiqué de condoléance destiné aux familles des deux premiers médecins décédés à la suite de leur contraction du coronavirus tout en précisant que la cause du décès des deux médecins n´est pas due à l'exercice de leurs fonctions professionnelles. Le Ministre du travail et de l'insertion professionnelle a récemment chargé une commission interne d´entreprendre une réflexion sur cette question. Qu´en est-il à ce jour, compte tenu de l´assise juridique marocaine, de la possibilité de reconnaître le caractère professionnel de la maladie provoquée par le coronavirus?

## Abstract (en)

Self-denial and sense of duty are fundamental ethical principles in health care. Since the outbreak of health crisis, healthcare workers have been the first bulwark against the spread of coronavirus, and therefore, the occupational category at higher risk of contamination. In this regard, in a statement dated 23 March 2020, the World Health Organization published a guidance regarding the management of the disease caused by Covid-19 in health workers, but also in workers employed in all sectors exposed to the risk of contamination. In Morocco, the Ministry of Health published on April 6, on its official website, a condolence message to the families of the first two doctors died following contraction of coronavirus, while specifying that coronavirus infection was not due to the exercise of their professional functions. The Minister of Labor and Professional Integration recently appointed an internal committee to undertake a reflection on this issue. At present, given Morocco’s law, what are the chances to categorize coronavirus as an occupational disease?

## Séminaire

### Introduction

Depuis le début de la crise sanitaire, le corps médical est le premier rempart contre la propagation du coronavirus. Tout le temps en contact avec des patients infectés ou suspects, ces soldats en blouse blanche peuvent tout autant être victimes de l´infection pandémique d´autant plus s´ils ne se sont pas suffisamment protégés. Le président du Conseil national de l´ordre des médecins au Maroc a révélé le 27 mars 2020 que 11 médecins ont été contaminés, sans suites pour ses valeurs qui auraient sans doute augmenté, le nombre total de professionnels de santé ayant attrapé la COVID-19 demeure inconnu. Le 6 avril, le ministère de la Santé a publié sur son site officiel un communiqué de condoléance destiné aux familles des deux premiers médecins décédés à la suite de leur contraction du coronavirus tout en précisant que la cause du décès des deux médecins n´est pas due à l'exercice de leurs fonctions professionnelles. La santé et la sécurité au travail constituent un droit fondamental de tous les travailleurs au Maroc. En effet, l´article 31 de la Constitution de 2011 garantit à tous l´accès aux conditions permettant de jouir des droits aux soins de santé, à la protection sociale, à la couverture médicale, à la solidarité et au travail. Qu´en est-il à ce jour, compte tenu de l´assise juridique marocaine, de la possibilité de reconnaître la maladie provoquée par le virus SARS-CoV-2 comme accident de travail ou maladie professionnelle ?

### Historique juridique de la santé au travail au Maroc

***1913:*** la première base juridique pour la réparation des accidents de travail prenait source du droit commun, l´article 750 du Dahir des obligations et des contrats DOC précise que : « il (l´employeur) répond également des accidents ou sinistres dont l´ouvrier, travaillant avec lui, est victime en exécutant le travail qui lui a été confié, lorsque l´accident ou le sinistre a pour cause la violation ou l´inobservation par l´employeur des règlements spéciaux relatifs à l´exercice de son industrie ou de son art », ainsi le salarié devait démonter la faute commise par son employeur devant le tribunal afin de pouvoir profiter d´une réparation de son dommage.

***1927 - 2002:*** la première législation spécifique, un peu plus protectrice du salarié qui est le Dahir du 25 Juin 1927, ayant institué un régime de responsabilité sans faute de l´employeur pour tous les dommages secondaires aux accidents de travail dont sont victimes les salariés, avec un système d´indemnisation forfaitaire. Ces dispositions qui étaient applicables exclusivement aux établissements industriels ont donné des retours positifs, ceci a favorisé leur élargissement à d´autres catégories professionnelles. En 1943, l´extension a visé -pour la première fois- les maladies professionnelles, suivie par un élargissement aux accidents de trajet en 1947. La responsabilité de l´employeur pouvait être couverte dans le cadre d´une assurance facultative. Les employeurs ayant choisi de ne pas recourir à l´assurance se chargeaient de la réparation sur leur patrimoine personnel. Ensuite la Loi n° 18-12 du 23 juillet 2002 a enfin apporté l´obligation d´assurance pour les accidents du travail.

***2015****:* actuellement les accidents du travail sont soumis à la Loi n° 18-12 publiée en janvier 2015. Cette loi a introduit une procédure moins longue et plus simple que les précédentes, comportant l´obligation de conciliation entre l´entreprise d´assurance et la victime, la révision de certaines indemnités et l´adaptation de la procédure civile. Elle définit les droits et obligations des parties prenantes et la procédure de déclaration mettant le directeur provincial de l´emploi en pierre angulaire des déclarations d´accidents de travail [1]. Concernant la liste des tableaux de maladies professionnelles, celle-ci est passée de 35 en 1972 à 95 en 1999 et enfin l´arrêté du 21 janvier 2014 l´a élargi à 111 tableaux cités dans le livre du professeur Ahmed Belhouss: « le droit médical : aspects déontologiques et juridiques de la relation médecin malade » Edition Media Events, Casablanca, 2018.

## COVID-19 et accident du travail

### L´accident de travail proprement dit en droit marocain

Il est défini dans l´article 3 du Dahir n° 1-14-190 du 22 janvier 2015 dans les termes suivants: « est considéré comme accident du travail, même si cet accident résulte d'un cas de force majeure ou si les conditions du travail ont mis en mouvement ou aggravé les effets des forces de la nature, à moins que l'employeur ou l'assureur ne rapporte la preuve d'une disposition pathologique de la victime, l'accident, quelle qu'en ait été la cause, survenu par le fait ou à l'occasion du travail à toute personne salariée ou travaillant à quelque titre ou en quelque lieu que ce soit, pour un ou plusieurs des employeurs ou chefs d'entreprises visés ci-dessous, ainsi le dommage dans le sens de cet article désigne toute lésion physique ou psychique causé par l´accident de travail et ayant entrainé une incapacité partielle ou totale, temporaire ou permanente ». À cet effet l´accident du travail doit rassembler les caractères suivants: se produire dans le cadre de l´activité professionnelle du salarié, sous le contrôle et l´autorité de l´employeur, en prenant en compte les temps de pause. Être soudain, contrairement à la maladie professionnelle et, entraîner une lésion physique ou psychique.

### L´accident de trajet

Selon l´article 4 de la Loi n° 18-12 12 relatives à la réparation des accidents du travail, promulguée par le Dahir n°1-14-190 n°6328 du 22 janvier 2015: c´est l´accident survenu à un travailleur pendant le trajet d´aller ou de retour, entre: le lieu du travail et sa résidence principale ou une résidence secondaire présentant un caractère certain de stabilité, ou tout autre lieu où le travailleur se rend d´une façon habituelle, Deuxièmement, entre le lieu du travail et le lieu où le travailleur prend habituellement ses repas, et entre ce dernier et sa résidence. Un détour pour un motif étranger aux nécessités du travail et de la vie courante n´est pas couvert par cette définition.

### L´accident dû à une faute intentionnelle ou inexcusable

Commise par la victime, la faute intentionnelle élimine le droit à la réparation du dommage selon l´article 309 du code de travail marocain. Or, quand il s´agit d´une faute intentionnelle commise par l´employeur ou ses préposés, selon l´article 310: la victime ou ses ayants droit conservent contre l´auteur de l´accident le droit de demander la réparation du préjudice causé conformément aux règles du droit commun ». Les articles 24 et 281 du code du travail marocain précisent que l´employeur est tenu de prendre les mesures nécessaires pour protéger la santé et la sécurité des travailleurs. Dans le contexte de la pandémie, cette obligation repose principalement sur les recommandations sanitaires émises par les autorités publiques. Enfin, s´il est prouvé que l´accident est dû à une faute inexcusable de la victime, le tribunal a le droit de diminuer la rente prévue qui lui a été attribuée selon le guide pratique de l´employeur publié en 2018 par la confédération générale des entreprises du Maroc.

### Accident du travail et la contamination par le SARS-CoV-2

Les modes de transmission de la fameuse infection font toujours sujets de discussion, mais il est principalement transmis sous forme de gouttelettes aéroportées disséminées par les éternuements ou la toux et le contact physique. Dans sa transmission, un individu infecté peut rester asymptomatique au début de l´infection, il s´introduit dans une population sensible, ainsi l´infection se propage [2]. En plus des éléments juridiques suscités, la jurisprudence marocaine exige la présence de témoins tout comme son analogue française (ex : audience publique N° 14-17691 du 18 juin 2015 de la Cour de cassation de la chambre civile, disponible sur Légifrance). Or pour le cas d´un virus de 30-kilo-base, ces exigences semblent hors de portée, d´autant plus si on considère la période d´incubation de 14 jours caractérisant le coronavirus, à moins qu´il s´agisse bien d´un évènement accidentel franc comme la projection accidentelle de secrétions bronchiques lors d´un geste d´intubation par exemple, mais ça ne pourrait pas représenter la majorité des cas de contaminations du personnel soignant au coronavirus.

Cependant, les prétentions suivantes peuvent jouer en faveur de la reconnaissance de l´origine professionnelle: d´abord si les règles édictées par le gouvernement relatives aux gestes de protection face à la pandémie n´ont pas été respectée par l´employeur, si l´employé a été en contact avec l´un de ses collègues reconnu COVID-19 positif, surtout en période de confinement, qui fut d´ailleurs précoce et prolongé au Maroc [3] et caractérisé par la limitation des activités autres que professionnelles, et enfin si l´on peut établir que personne n´a contracté le virus dans l´entourage privé du salarié [4]. Il est aussi recommandé aux employés souhaitant déclarer leur contamination au coronavirus comme accident de travail, d´obtenir un certificat médical confirmant le diagnostic et les résultats du test de dépistage. La date de ce teste pourrait être considérée à titre approximatif comme date d´accident de travail.

### Particularité procédurale de la crise sanitaire

Initialement, la victime d´accident de travail ou ses ayants droit devaient informer l'employeur le jour même. Depuis le 15 janvier 2015, ce délai est prolongé à 48h sauf cas de force majeure, récemment, l´article 6 du décret-loi n° 2-20-292 édictant des dispositions particulières à l´état d´urgence sanitaire stipule que : « tous les délais prévus par les textes législatifs et réglementaires en vigueur sont suspendus pendant la période de l´état d´urgence sanitaire déclaré. Ils recommencent à courir à compter du lendemain de la levée de l´état d´urgence précité ».

## COVID-19 et maladie professionnelle

### La reconnaissance juridique de la maladie professionnelle

Une maladie est “professionnelle” si elle est la conséquence directe de l'exposition d'un travailleur à un risque physique, chimique, biologique, ou résulte des conditions dans lesquelles il exerce son activité professionnelle, la liste des maladies professionnelles au Maroc comporte 111 tableau selon l´arrêté ministériel du 21 janvier 2014 (modifiant et complétant l´arrêté ministériel du 23 décembre 1999), ces tableaux comportent l´énumération, rangée par rubrique des conditions que l´employé doit remplir, pour bénéficier de la présomption d´origine professionnelle sans qu´il n´ait besoin d´apporter des preuves. La liste actuelle des maladies professionnelles (MP) causées par des agents biologiques infectieux ou parasitaires comporte 19 tableaux, nous citons : brucelloses, tétanos, charbon, spirochétoses (à l´exception des tréponématoses), pasteurelloses, kérato-conjonctivites virales, ornithose psittacose, poliomyélites, maladies dues aux bacilles tuberculeux et à certaines mycobactéries atypiques.

Dans le tableau n° 3.10, nous avons: « les maladies liées à des agents infectieux ou parasitaires contractées en milieux d'hospitalisation et d'hospitalisation à domicile », ce tableau comporte respectivement: les infections dues aux staphylocoques, *Pseudomonas aerugioosa*, pneumocoques, streptocoques béta-hémolytique, méningocoques, fièvres typhoïde et paratyphoïde, dysenterie bacillaire, choléra, fièvres hémorragiques, gonocoques, syphilis, infections à herpès virus, gale. Dans la case: « staphylocoque », il est indiqué : « tous les travaux accomplis par le personnel de soins et assimilé, de service, d´entretien, ou de services sociaux mettant au contact d´un réservoir de staphylocoque ». L´infection due au coronavirus ne figure pas sur les listes de maladies professionnelles puisqu´il s´agit d´une maladie infectieuse émergente dont les données sont toutes récentes. Cependant, si on garde le même mode de raisonnement, les travaux du personnel de soins en période pandémique les mettent au contact d´un réservoir COVID-19.

C´est pourquoi, l´introduction cette infection aux tableaux des maladies professionnelles pour le personnel soignant s´avère primordial. Il faudrait également le généraliser à toutes les professions essentielles ayant assuré la continuité des services sous le risque de contamination.

### Pour les employés du secteur public

On parle de « maladie contractée au service » dans l´article 45 du code de la fonction publique stipulant ce qui suit: « lorsque la maladie est contractée ou aggravée dans l'exercice ou à l'occasion de l'exercice des fonctions, soit en accomplissant un acte de dévouement dans un intérêt public ou pour sauver la vie d'une ou plusieurs personnes, soit à la suite d'un accident survenu dans l'exercice ou à l'occasion de l'exercice des fonctions, le fonctionnaire reçoit l'intégralité de ses émoluments jusqu'à ce qu'il soit en état de reprendre son service ou jusqu'à ce qu'il soit reconnu définitivement inapte et admis à la retraite dans les conditions prévues par la Loi n° 011-71. Le fonctionnaire a droit, au remboursement par l'administration des honoraires médicaux et des frais entraînés directement par la maladie ou l'accident » (Dahir n° 1.58.008 du 4 chaabane 1377 portant Statut Général de la Fonction Publique). Or, le dévouement pour la sauvegarde de la vie humaine a bien caractérisé l´activité des professionnels de soins pendant cette crise sanitaire. Sur le plan administratif pratique, la circulaire du Premier Ministre n°10/2018 [5] évoque la notion de « maladies et accidents attribués au travail », ainsi il confère à la commission de réforme et au « conseil de santé » émanant du ministère de la Santé la décision de reconnaissance du caractère professionnel de la maladie ne figurant pas dans les tableaux.

### Pour les employés du secteur privé

L´arrêté du ministre du travail et des affaires sociales n°100-68 du 20 mai 1967, réglemente l´indemnisation des maladies professionnelles figurant dans les tableaux, mais il ajoute la notion de « maladies ayant un caractère professionnel ». L´arrêté du ministre de l´emploi et de la formation professionnelle n°2625-12 du 16 juillet 2012 reconnait la possibilité de survenue, et tacitement d´indemnisation de maladies qui pourraient être d´origine professionnelle. Dans le même sens, les magistrats de la Cour de cassation de Rabat ont estimé que la liste des maladies inscrites dans les tableaux n´était pas limitative. Ils ont ainsi élargi le champ de prise en charge aux pathologies, non visées par ceux-ci, mais déclarées par le médecin traitant ou le médecin du travail, comme ayant une origine professionnelle.

La littérature a documenté des cas où l´infection virale été impliquée dans la contamination des soignants, surtout en chirurgie. Il s´agit du virus de l´immunodéficience humaine (VIH), du virus de l´hépatite B, du virus du papillome bovin et du virus du papillome humain (VPH). Selon Calero *et al*.un cas de papillomatose laryngée fut reconnu comme maladie professionnelle en Allemagne chez une infirmière de 28 ans, ayant participé à plusieurs gestes d´excisions électro-chirurgicales et lasers des condylomes ano-génitaux, elle a développé une papillomatose laryngée récurrente et prouvée histologiquement. L'avis d'expert d'un institut virologique a confirmé une forte probabilité de corrélation entre l'exposition professionnelle et la papillomatose laryngée de sorte qu'elle a été acceptée comme maladie professionnelle [6]. Ainsi la jurisprudence, en collaboration avec les experts techniques, peuvent réussir à donner un cadre législatif pour la reconnaissance et la réparation, de la COVID-19 d´origine professionnelle pour les travailleurs de tous les secteurs.

### L´expertise médicale en réparation de maladie professionnelle/accident de travail

Le recours à une expertise en matière de réparation d´AT/MP est régi par les dispositions des articles 55 à 66 du code de la procédure civile dans sa version révisée en date du 6 juin 2013, il obéit à la règle du contradictoire, son but principal est de statuer sur l´imputabilité des séquelles -constatées par l´expert- à la sinistralité objet de l´expertise. L´expert doit d´abord établir l´historique professionnel du salarié, vérifier la réalité de l´exposition aux risques professionnel sujet de l´expertise, et se documenter sur: les tâches exécutées, les conditions des locaux et les moyens de prévention utilisés. Dans l´« exposé des faits » faisant partie du rapport d´expertise, il serait important de décrire succinctement, à partir des récit et des documents fournis, les conditions de travail ayant probablement permis la contamination, par exemple: le non-respect des règles de distanciation et des gestes barrières, la promiscuité du lieu de travail, le contact prolongé et rapproché avec la clientèle ou avec un collègue dont la contamination a été confirmée, l´absence des équipements de protection individuelle ou de matériel sanitaire disponible. En cas d´existence d´un état antérieur, l´expert tâchera d´expliquer s´il a interféré, et dans quelle mesure, avec les lésions actuelles [7], dans la conclusion l´expert précise le taux d´incapacité, la nécessité de changement d´emploi et la nécessité d´assistance d´une tierce personne.

### La COVID-19 professionnelle en droit comparé

L´OMS a publié le 23 mars 2020 son rapport de conférence pour la gestion de l´épidémie de COVID-19 en milieu de travail, elle a défini 4 niveaux de risque pour toutes les catégories professionnelles et où les professionnels de santés ont été classés comme « travailleurs à très haut risque ». Dans ce rapport l´OMS a souligné le rôle des services de santé au travail dans l´évaluation du risque, l´identification des groupes de travailleurs à risque, le développement des plans de prévention, le conseil sur la santé au travail, mais aussi leur rôle à aider les salariés à faire valoir leurs droits à la reconnaissance en maladie professionnelle ou en accident du travail [8]. En fonction des pays, la reconnaissance et la prise en charge de la COVID-19, comme sinistralité au travail est variée.

### Italie

L´Institut national d'assurance contre les accidents industriels (INAIL) d´Italie, a confirmé que l'infection au coronavirus des médecins, infirmiers et autres employés du Service national de santé est considérée comme une maladie professionnelle. Pour ce groupe d'employés, le lien de causalité entre le travail et l'infection est automatiquement supposé, ceci est dans le but de fournir la couverture pour les cas où il est difficile de déterminer les causes spécifiques et les méthodes de travail liées à l'infection. De plus, la protection de l'INAIL s'applique également aux cas de COVID-19 dans lesquels le virus SARS-CoV-2 a été contracté sur le chemin du travail [9].

### France

Dans les cas où les critères du tableau de maladies professionnelles ne sont pas remplis, le dossier est étudié par des médecins experts des pathologies d´origine professionnelle. Réunis au sein du «comité régional de reconnaissance des maladies professionnelles » (CRRMP), ces experts se prononcent sur le lien entre le travail et la pathologie et leur avis s´impose à la caisse primaire d´assurance maladie (CPAM), le ministre des Solidarités et de la santé en France a annoncé le 23 mars 2020 que l´infection à la COVID-19 serait systématiquement et automatiquement reconnue comme maladie professionnelle pour les soignants et pour certaines catégories de personnel exerçant leur activité professionnelle dans des services déterminés.

### Espagne

Initialement, tous les arrêts de travail dus à la COVID-19 étaient considérés comme arrêt pour maladie simple, avec une exception selon l´article 5 du décret-loi royal 6/2020 du 10 mars : « ils seront considérés, à titre exceptionnel, comme une situation assimilée à un accident du travail, exclusivement au profit financier d'une invalidité temporaire de la sécurité sociale, ces périodes d'isolement ou de contagion des travailleurs causées par le virus COVID-19 ». Ensuite, le décret-loi royal 13/2020 du 8 avril 2020 (par le Journal officiel de l'État, 8 avril 2020, n° 9, pages 28524-28542), a modifié l'article 5 : en plus de maintenir la considération comme situation assimilée à un accident du travail (AT), il introduit la possibilité d'être classée comme AT quand il est prouvé que la contagion de la maladie a été contractée exclusivement pour la performance du travail dans les termes indiqués à l´article 156 du texte consolidé de la loi générale de sécurité sociale espagnole. Martí-Amengual *et al*. estiment que pour le personnel de santé cette infection ne devrait pas être considérée comme maladie courante et même pas comme AT, mais comme maladie professionnelle [10].

### Tunisie

Étant une maladie hors tableaux, aucune indemnisation à titre de maladie professionnelle ne pourra être envisagée avec la règlementation actuelle en Tunisie. En la qualifiant d´un accident de travail assimilé aux accidents d´exposition au sang et aux liquides biologiques, l´employé prouvé COVID-19 positif, a droit à des prestations temporaires en nature (les soins) et en espèces (indemnités pour compenser l´incapacité de gain dans la période d´arrêt de travail). Dans le secteur privé, l´indemnisation de l´accidenté commence à partir du quatrième jour suivant l´accident, elle correspond aux 2/3 du salaire global journalier. Dans le secteur public, la victime de d´AT conserve l´intégralité de sa rémunération ainsi que ses droits à l´avancement et à la promotion selon le *Groupement de Médecine du Travail De Sfax*.

## Conclusion

Au Maroc, la situation actuelle ne permet pas de réparation pour le salarié ou ses ayants droit en cas de séquelles ou de décès relatif à l´infection due au coronavirus. Si sa reconnaissance en tant que maladie professionnelle ou accident de travail n´est pas encore assurée, le « Ministre du Travail et de l'insertion professionnelle » a récemment chargé une commission interne d´entreprendre une réflexion sur cette question. Il serait logique que cette reconnaissance se distingue en fonction des secteurs de travail où l´origine professionnelle semblerait plus évidente comme il a été établi en Italie. Le secteur médical est placé en tête de liste. La simplification des procédures administratives s´avère primordiale, elle permettrait d´améliorer le contexte de sous-déclaration des sinistralités professionnelles que connait le pays.
